# Diagnostic value of fecal calprotectin in primary care patients with gastrointestinal symptoms: A retrospective Swedish cohort study

**DOI:** 10.1002/jgh3.12972

**Published:** 2023-09-22

**Authors:** Zlatica Rendek, Magnus Falk, Ewa Grodzinsky, Stergios Kechagias, Henrik Hjortswang

**Affiliations:** ^1^ Department of Biomedical and Clinical Sciences Linköping University Linköping Sweden; ^2^ Department of Health, Medicine and Caring Sciences Linköping University Linköping Sweden; ^3^ Primary Health Care Centre Kärna Linköping University Linköping Sweden; ^4^ Division of Diagnostics and Specialist Medicine, Department of Health, Medicine and Caring Sciences Linköping University Linköping Sweden; ^5^ Department of Gastroenterology and Hepatology in Linköping, and Department of Health, Medicine, and Caring Sciences Linköping University Linköping Sweden

**Keywords:** fecal calprotectin, gastrointestinal disease, inflammatory bowel disease, nonsteroidal anti‐inflammatory drugs, proton pump inhibitors

## Abstract

**Aims:**

To investigate the diagnostic accuracy of fecal calprotectin (FC) for inflammatory bowel disease (IBD) and organic gastrointestinal disease (OGID) in primary care. To examine the association with demographic factors, symptoms and concomitant medical therapy.

**Methods:**

A retrospective analysis of data on all semiquantitative FC tests from individuals ≥18 years conducted in primary care in Östergötland County in 2010. A 5‐year follow‐up with inclusion of new gastrointestinal diagnoses.

**Results:**

A total of 1293 eligible patients were included. IBD was found in 8.8% and other OGID in 30.8% of patients with positive FC. Positive FC was associated with diarrhea, age >60 years, duration <3 months, use of nonsteroidal anti‐inflammatory drug (NSAID), and proton pump inhibitor (PPI). Predictors of IBD were positive FC, diarrhea, rectal bleeding, and male sex; predictors of OGID positive FC, age >35 years, abnormal clinical findings, and duration <3 months. FC yielded the highest sensitivity and negative predictive value compared with demographic factors, symptoms, and duration. Use of NSAID and PPI showed a marginal increase in the sensitivity, positive predictive value, and decrease in the specificity of FC. Within 5 years, 4.0% had a new gastrointestinal diagnosis among patients with positive FC (0.6% IBD).

**Conclusions:**

FC reliably rules out IBD and contradicts the presence of other OGID in primary care patients. Positive FC test together with other predictors, such as diarrhea, rectal bleeding, short duration, or age >35 years, should encourage a prioritized investigation. Use of NSAID, PPI, and ASA may affect the diagnostic accuracy of FC for IBD and OGID.

## Graphical abstract

Fecal calprotectin reliably rules out IBD and contradicts the presence of other OGID in primary care patients. Treatment with nonsteroidal anti‐inflammatory drugs and proton pump inhibitors may affect the diagnostic accuracy of fecal calprotectin.

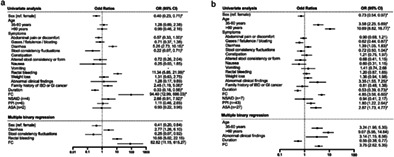

## Introduction

Patients with gastrointestinal (GI) symptoms represent approximately 10% of primary care appointments and are a frequent diagnostic challenge for general practitioners (GPs).^1^, ^2^ GPs need to differentiate between functional and organic GI diseases in order to decide whether further investigations are necessary. Symptoms are often nonspecific and may not allow for this distinction. Patients are frequently referred to specialist care for further examinations. These include endoscopic procedures, which are invasive and require bowel preparation, in addition to the risk of severe complications. Previous studies of patients referred to secondary care due to intestinal symptoms only revealed an organic diagnosis in 13–37%.^3^, ^4^, ^5^, ^6^ The need to support GPs in the management of patients with GI symptoms has been advocated for many years.^7^, ^8^

Fecal calprotectin (FC) is reported to be a reliable marker of intestinal inflammation. It is a useful tool for differentiating between inflammatory bowel disease (IBD) and irritable bowel syndrome (IBS), and monitoring of disease activity in IBD patients.^9^, ^10^ However, elevated FC has been ascribed to several other factors, including treatment with nonsteroidal anti‐inflammatory drugs (NSAIDs), proton pump inhibitors (PPIs), or acetylsalicylic acid (ASA).^6^, ^11^, ^12^, ^13^ Previous studies have confirmed elevated FC in 32–57% of the patients with normal colonoscopy^11^, ^14^, ^15^ and found an association with these drugs.^11^

The measures of FC test accuracy have mainly been performed in selected secondary care populations, and the evidence available from primary care is still limited.^7^, ^16^, ^17^ Freeman *et al*.^18^ have recently highlighted the problem of an inconsistent FC testing in primary care, referring to the small size and heterogeneousness of available studies. A care pathway for the FC use in primary care, developed in United Kingdom to optimize the differentiation between IBS and IBD, was shown to lead to a reduction in referrals and cost savings.^19^, ^20^ Parameters such as sensitivity and negative predictive value (NPV) can assist GPs in determining which patients are eligible for referral. However, FC has mostly been assessed as an individual test without accounting for other factors.^9^, ^10^, ^21^

The aim of the present study was to determine the diagnostic accuracy of FC for IBD and organic GI disease (OGID) in primary care patients with gastrointestinal symptoms and to examine the association with demographic factors, symptoms, and concomitant medical therapy.

## Material and methods

### Data collection

Data on all semiquantitative FC tests analyzed in Östergötland County in 2010 were retrieved from the Department of Clinical Chemistry, Center for Diagnostics, Linköping University Hospital, Sweden. At that time, just over 343 000 people ≥18 years lived in the county.^22^ Only tests from individuals ≥18 years conducted in primary care were included. In patients with multiple tests, the first test that initiated the investigation was included, regardless of whether it was higher or lower than subsequent tests. Patients with missing medical records or previously known GI diagnosis of IBD or GI cancer were excluded.

Medical records were retrospectively reviewed in 2013, with focus on patients' GI problems and investigation results. Data collected were sex, age, symptoms, and symptom duration (according to Table 1), which were documented during a GP appointment in connection with FC test. Concomitant medical therapy (on the basis of subgroups of NSAIDs, PPIs and ASA) was recorded, as well as the final diagnosis registered by the GP or, in case of referral, by a gastroenterologist. In Swedish healthcare, diagnosis coding based on International Classification of Diseases (ICD‐10) is registered in the patient's medical record at every doctor visit or hospital stay, so the final diagnosis could be collected retrospectively. Data were systematically entered into a table with qualitative field notes based on the patient's record review made by one investigator (Z.R.).

**Table 1:** Clinical characteristics of participants with negative and positive fecal calprotectin (FC)

	FC < 15 mg/kg, *n* (%)	FC ≥ 15 mg/kg, *n* (%)	*P* values^†^	OR (95% CI)^‡^
Sex			0.112	
Male	232 (32.4)	212 (36.7)		
Female (ref.)	483 (67.7)	366 (63.3)		
Age			**0.000**	
<35 years (ref.)	309 (43.2)	158 (27.3)		
35–60 years	264 (36.9)	200 (34.6)		^b^
>60 years	142 (19.9)	220 (38.1)		2.59 (1.87, 3.58)
Symptoms				
Abdominal pain or discomfort	461 (64.5)	371 (64.2)	0.953	
Gases / flatulence / bloating	205 (28.7)	153 (26.5)	0.382	
Diarrhea	270 (37.8)	271 (46.9)	**0.001**	1.56 (1.20, 2.01)
Stool consistency fluctuations	146 (20.4)	115 (19.9)	0.835	
Constipation	57 (8.0)	44 (7.6)	0.836	
Altered stool consistency or form	69 (9.7)	56 (9.7)	1.000	
Nausea	64 (9.0)	20 (3.5)	**0.000**	0.35 (0.19, 0.64)
Vomiting	30 (4.2)	21 (3.6)	0.668	
Rectal bleeding	166 (23.2)	164 (28.4)	**0.040**	^b^
Weight loss	97 (13.6)	93 (16.1)	0.207	
Abnormal clinical findings	16 (2.2)	12 (2.1)	1.000	
Family history of GI cancer or IBD	67 (9.4)	49 (8.5)	0.625	
Duration			**0.000**	0.59 (0.45, 0.77)
<3 months	168 (23.5)	196 (33.9)		
>3 months	439 (61.4)	288 (49.8)		
Unclear	108 (15.1)	94 (16.3)	^a^	
Concomitant medical therapy				
NSAID	13 (1.8)	28 (4.8)	**0.002**	2.72 (1.32, 5.62)
PPI	48 (6.7)	106 (18.3)	**0.000**	2.92 (1.93, 4.41)
ASA	28 (3.9)	42 (7.3)	**0.009**	^b^

*Note*: Statistically significant results at *P* <0.05 are in bold.

^†^ Univariate analysis performed with Fisher's exact test, except for age, which was conducted using Pearson's chi‐squared test.

^‡^ Binary logistic regression. The dependent variable is FC.

^a^ Unclear duration was removed from the relevant statistical analysis.

^b^ Statistically nonsignificant variables removed from the model.

Each patient could have reported more than one symptom.

ASA, acetyl salicylic acid; CI, confidence interval; GI, gastrointestinal; IBD, inflammatory bowel disease; NSAID, nonsteroidal anti‐inflammatory drug; OR, odds ratio; PPI, proton pump inhibitor.

In cases of multiple findings, two investigators (Z.R. and H.H.) thoroughly reviewed patients' medical records and chose the significant diagnosis, that is, that which correlated clinically with patients' symptoms and examination results. IBD and tumor diagnoses were prioritized. For example, if the investigation revealed ulcerative colitis (UC) and gastroesophageal reflux disease in a patient presenting with diarrhea and rectal bleeding, UC was chosen.

Subsequently, a follow‐up was performed, by retrieving all records from Östergötland County between 2010 and 2015, pertaining to the ICD‐10 diagnosis code groups C‐D (Neoplasms) and K (Diseases of the digestive system). This aimed to identify any possible missed cases of IBD or OGID in patients with a negative FC test, in whom the GPs did not proceed with further investigation. Patients were matched to those included in our study and only diagnoses related to the GI tract were selected. Diagnoses made in 2010 were excluded, as these were already reviewed during the primary analysis. Diagnoses were compared with previous results of investigations as well as patients´ known GI conditions and only new GI findings were included. The follow‐up took place during the time the patients lived in Östergötland or until death.

### Fecal calprotectin analysis

FC was analyzed with the semiquantitative immunochromatographic rapid test PreventID® CalDetect® (Preventis, Luxemburg) at the Department of Clinical Chemistry, Center for Diagnostics, Linköping University Hospital. It is an immunological lateral flow test for the detection of human calprotectin via gold‐conjugated anti‐calprotectin antibodies. The results are expressed as FC concentrations of <15 mg/kg, 15–60 mg/kg, or >60 mg/kg. According to the manufacturer, the test is considered positive when the concentration is ≥15 mg/kg.^23^

### Statistical analyses

Statistical analyses were performed using SPSS version 27.0 (IBM, Armonk, NY, USA). Data are presented as numbers, percentages, or median, range and interquartile range (IQR). Age was divided into three categories and hence regarded as a categorical variable. Fisher's exact test or Pearson's chi‐squared test was used for nominal data to compare proportions. Clinical variables that showed a *p* value <0.10 were included in a binary logistic regression model. Predictors of IBD and OGID selected in the binary logistic regression were assessed for accuracy (sensitivity, specificity, NPV, and positive predictive value [PPV]). Confidence intervals for sensitivity, specificity, NPV, and PPV were calculated using the method described by Newcombe with continuity correction^24^; *p* value <0.05 was considered statistically significant.

### Ethical approval

The Regional Ethical Review Board approved the study protocol (Dnr 2011/467–31) and subsequently the amendment pertaining to a 5‐year follow‐up (Dnr 2016/456–32).

## Results

### Sample characteristics

**Figure 1:**
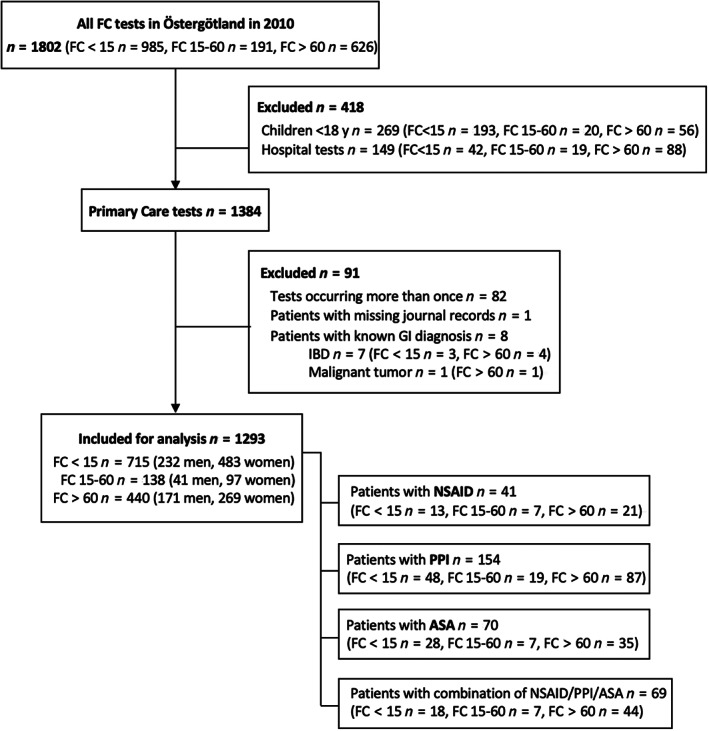
Study flow diagram. FC, fecal calprotectin; GI, gastrointestinal; IBD, inflammatory bowel disease; NSAID, nonsteroidal anti‐inflammatory drug; PPI, proton pump inhibitor; ASA, acetyl salicylic acid.

The total number of FC tests analyzed was 1802. Patients aged <18 years (*n* = 269) and tests that had not been conducted in primary care (*n* = 149) were excluded. Subsequently, FC tests in patients occurring more than once (*n* = 82), patients with missing journal records (*n* = 1), and previously known diagnosis of IBD or GI cancer (*n* = 8) were also excluded. Finally, 1293 eligible patients were included (median age, 43 years; range, 18–93 years; IQR, 34 years) (Fig. 1). Of these, 715 had a negative FC test (median age, 37 years; range, 18–93 years; IQR, 30 years) and 578 had a positive FC test (median age, 52 years; range, 18–92 years; IQR, 34 years).

There were 41 patients on NSAID (3%; median age, 53 years; range, 19–87 years; IQR, 22 years), 154 patients on PPI (12%; median age, 54 years; range, 18–91 years; IQR, 29 years), 70 patients on ASA (5%; median age, 71.5 years; range, 35–93 years; IQR, 14 years), and 69 patients on combination of these (5%; median age, 67 years; range, 18–90 years; IQR, 23 years).

### Positive versus negative FC test

Distribution of patient characteristics according to FC outcome is presented in Table 1 and Supplementary Table S1. Patients with diarrhea (OR = 1.56) and age > 60 years (OR = 2.59) had a higher likelihood of a positive FC. In contrast, patients with nausea (OR = 0.35) and duration >3 months (OR = 0.59) had a lower likelihood of a positive FC. Statistically significant associations with rectal bleeding and age 35–60 years were seen in the univariate analysis but found to be nonsignificant in a binary logistic regression. Most patients experiencing diarrhea and rectal bleeding reported duration >3 months (57.7% and 53.9%, respectively), compared with those who reported <3 months (32.7% and 36.1%, respectively).

The use of NSAID, PPI, and ASA was associated with a positive FC in the univariate analysis (*P* < 0.05); however, only NSAID and PPI were found to be significant in the binary logistic regression.

### Results of investigations and prediction models of IBD and OGID

Tables 2 and Supplementary Table S2 present the outcome of the GI investigations following FC testing. Altogether, 39.6% of patients with positive FC had an organic GI disease (*n* = 229; IBD, other GI inflammation, GI infections and GI tumors as presented in Table 2), compared with 9.7% of patients with negative FC (*n* = 69). Of those with positive FC, 8.8% were diagnosed with IBD and 30.8% with OGID (which includes other GI inflammation, GI infections and GI tumors), compared with 0.1% and 9.5%, respectively, of those with negative FC tests. In contrast, a higher proportion of patients with negative FC had functional GI disorders (FGIDs; 33.6% vs 22.7%) and other diagnoses in the GI tract.

**Table 2:** Final diagnoses

	FC <15 mg/kg (*n* = 715)		FC 15–60 mg/kg (*n* = 138)	FC >60 mg/kg (*n* = 440)	
	n	%	n	n	%^†^
IBD (*n* = 52)		0.1			8.8
Crohn's disease^‡^	1		0	12	
Ulcerative colitis	0		2	36	
IBD unclassified	0		0	1	
Other GI inflammation (*n* = 168)		6.3			21.3
Microscopic colitis	2		2	13	
Gastritis	3		0	3	
Gastric ulcer	1		0	1	
*Helicobacter pylori* infection	2		1	4	
Gastroesophageal reflux disease with esophagitis	0		0	2	
Diverticulitis/diverticulosis	28		8	72	
Nonspecific proctitis	2		0	2	
Celiac disease	4		0	2	
Other GI inflammatory conditions	3		1	12	
GI infections (*n* = 32)	12	1.7	4	16	3.5
GI tumors (*n* = 46)		1.5			6.1
Benign (polyps)	5		2	10	
Malignant^§^	6		3	20	
Functional GI disorders (FGIDs) (*n* = 371)		33.6			22.7
Irritable bowel syndrome	186		34	63	
Dyspepsia	4		1	0	
Constipation	6		2	4	
Functional diarrhea	32		4	19	
Unspecified functional bowel disorder	12		3	1	
Other diagnoses in the GI tract (*n* = 96)		10.1			4.2
Lactose intolerance	8		2	2	
Gallstones	6		0	0	
Gastroesophageal reflux disease without esophagitis	10		1	3	
Miscellaneous macroscopic abnormalities of the gastric mucosa	3		0	0	
Anorectal disease	45		7	9	
Other diagnoses and conditions (*n* = 35)	24	3.4	5	6	1.9
Normal findings (*n* = 493)	310	43.4	56	127	31.7

^†^ Data combined for FC 15–60 mg/kg and >60 mg/kg. IBD, inflammatory bowel disease; GI, gastrointestinal.

^‡^ Crohn's disease (according to the Montreal classification): FC ≥15 mg/kg: A2, 17–40 years, *n* = 5; A3, >40 years, *n* = 7; L1, ileal, *n* = 3; L2, colonic, *n* = 5; L3, ileocolonic, *n* = 3; L1L4, ileal and upper GI tract, *n* = 1; B1, non‐stricturing/non‐penetrating, *n* = 11; B3p, penetrating perianal disease, *n* = 1. FC <15 mg/kg: A3, L3, B1.

^**§**^ Malignant tumors: colorectal cancer (*n* = 15), polyps with high‐grade dysplasia (*n* = 7), pancreatic cancer (*n* = 3), small bowel adenocarcinoma (*n* = 1), liver cancer metastasized (*n* = 1), cholangiocarcinoma (*n* = 1), intestinal T‐cell lymphoma (*n* = 1).

In patients diagnosed with Crohn's disease (*n* = 13), according to the Montreal classification, the most common age group was >40 years (61.5%), colonic disease (38.5%), and non‐stricturing and non‐penetrating behavior (92.3%) (Table 2).

**Figure 2:**
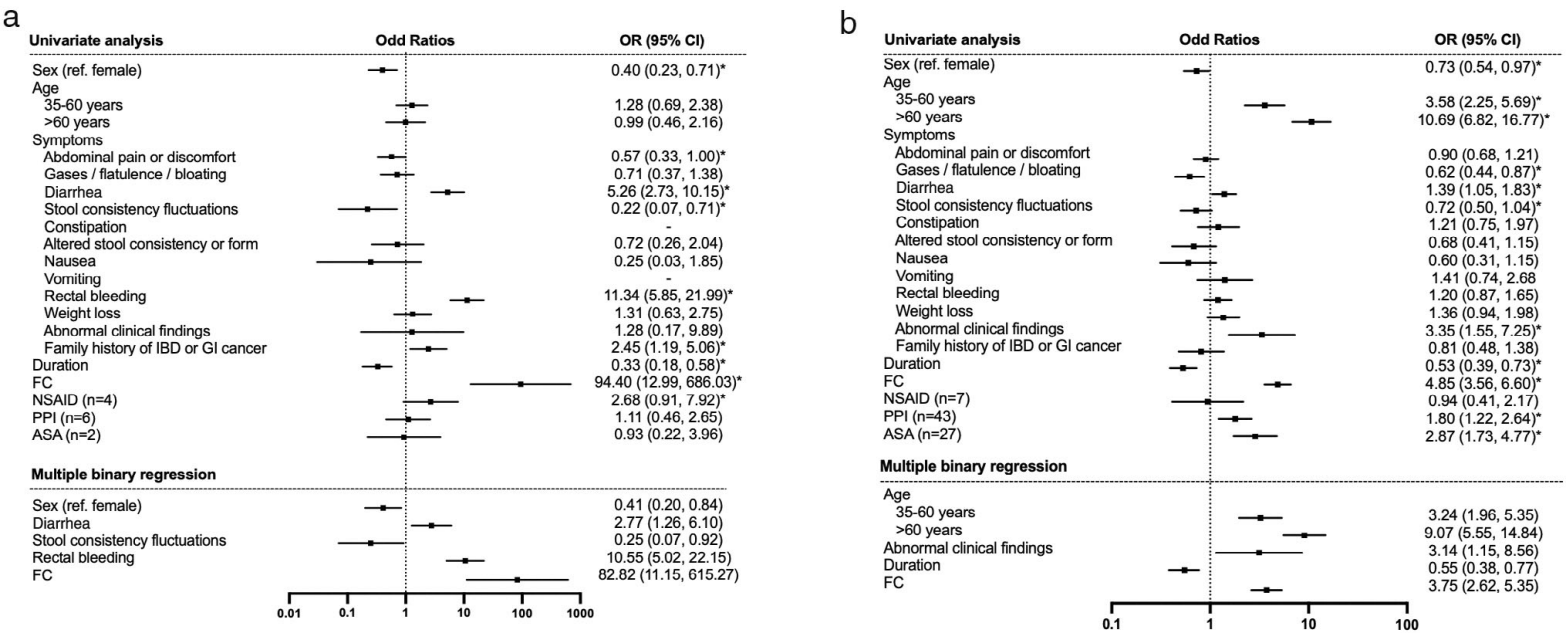
Forest plots off odds ratios (OR) based on logistic regression with dependent variable (a) inflammatory bowel disease (IBD) and (b) organic gastrointestinal disease (OGID) and independent variables sex, age, symptoms, duration, FC, and drugs. *Variables included in binary logistic regression. CI, confidence interval; GI, gastrointestinal; FC, fecal calprotectin; NSAID, non‐steroidal anti‐inflammatory drug; PPI, proton pump inhibitor; ASA, acetyl salicylic acid.

Associations between IBD, OGID, and clinical outcome variables were further explored statistically (Fig. 2 and Supplementary Table S3). The likelihood of IBD was higher in patients with positive FC (OR = 82.82), diarrhea (OR = 2.77), and rectal bleeding (OR = 10.55) but lower in females (OR = 0.41) and patients with stool consistency fluctuations (OR = 0.25). A statistically significant association with family history of IBD or GI cancer and duration was seen in the univariate analysis (*P* < 0.05) but found to be nonsignificant in the binary logistic regression. Abdominal pain was more commonly found in negative FC patients (*P* < 0.05) and apart from that, it did not have any statistical significance.

OGID was associated with positive FC (OR = 3.75), higher age (35–60 years, OR = 3.24; >60 years, OR = 9.07), and abnormal findings in abdominal physical examination (OR = 3.14). The likelihood of OGID was lower in patients with duration >3 months (OR = 0.55). Significant association with sex, gases, diarrhea, and stool consistency fluctuations was seen for OGID in the univariate analysis (*P* < 0.05) but not in the binary logistic regression.

NSAID use was more commonly found in patients with IBD, PPI, or ASA use in patients with OGID (*P* < 0.05). However, none of the drugs showed significant associations with IBD or OGID by binary logistic regression (Fig. 2).

### Diagnostic accuracy measures

**Figure 3:**
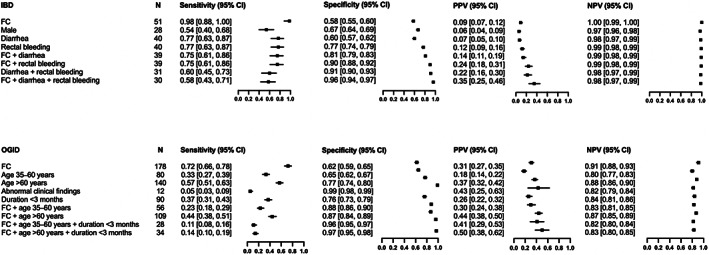
Diagnostic accuracy of fecal calprotectin (FC) and other variables for inflammatory bowel disease (IBD) and organic gastrointestinal disease (OGID). Only selected significant factors predicting the respective disease by means of binary logistic regression are included. CI, confidence interval; PPV, positive predictive value; NPV, negative predictive value; GI, gastrointestinal.

The diagnostic accuracy of potential predictors of IBD and OGID is summarized in Figure 3. FC yielded the highest sensitivity and NPVs among other variables studied. In general, adding other significant predictors increased the specificity and PPV, but decreased the sensitivity. Abdominal pain (not presented in Fig. 3) was found to be nonspecific and including it in analysis of IBD decreased the sensitivity and PPV but increased the specificity only marginally.

**Figure 4:**
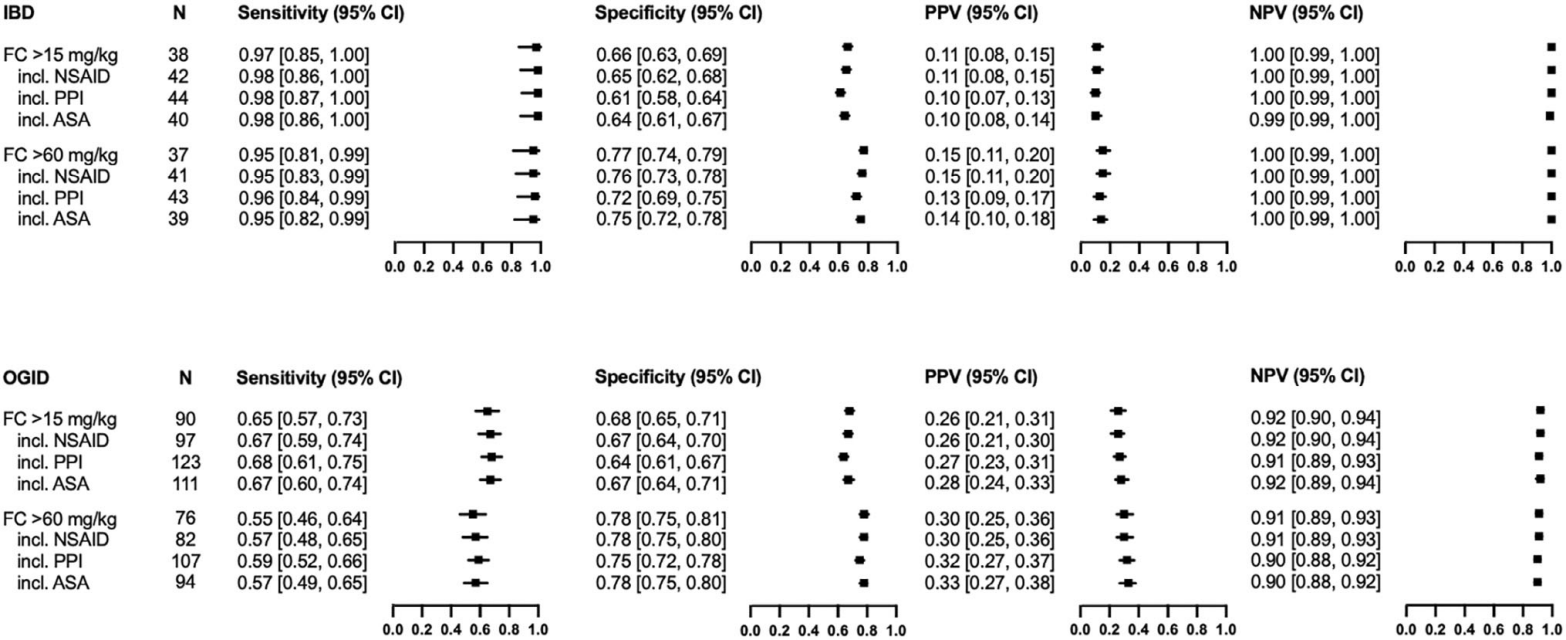
Diagnostic accuracy of fecal calprotectin (FC) at a cutoff of 15 and 60 mg/kg for inflammatory bowel disease (IBD) and organic gastrointestinal disease (OGID) and the influence of a concomitant medical therapy on the measures of the test accuracy. CI, confidence interval; PPV, positive predictive value; NPV, negative predictive value; NSAID, non‐steroidal anti‐inflammatory drug; PPI, proton pump inhibitor; ASA, acetyl salicylic acid.

Figure 4 presents the diagnostic accuracy of FC for IBD and OGID. Increasing the cutoff value from 15 to 60 mg/kg demonstrated an increase in the specificity by 10% and PPV by 4%, and a decrease in the sensitivity by 2–10%. NPV remained essentially unchanged. Use of NSAID, PPI, and ASA showed only minor changes in the accuracy measures of FC, in particular a tendency to increase the sensitivity, PPV, and decrease the specificity. However, the NPV value was not specifically affected.

### Five‐year follow‐up

For the purpose of the follow‐up, 8083 records were retrieved. Ninety‐two patients (7.1%) moved to another county and were lost to follow‐up. New GI diagnoses were found in 78 patients (FC < 15 *n* = 36, FC 15–60 *n* = 5, FC > 60 *n* = 37; Supplementary Table S4). Among patients with a positive FC test, three (0.6 valid percent) developed IBD and four (0.7 valid percent) developed a malignant GI tumor. In the negative group, no patient developed IBD and three (0.5 valid percent) were diagnosed with malignant tumors. Generally, new organic GI disease was diagnosed in 4.0 valid percent of patients with positive FC compared with 3.1 valid percent of patients with negative FC.

## Discussion

The present study reports on the diagnostic measures of FC in a large primary care population with GI symptoms and associations with demographic factors and symptom combinations that enable a positive diagnosis. FC showed higher sensitivity and NPV than other demographic factors and symptoms for predicting IBD and OGID. Combining significant predictors with FC increased specificity and PPV but tended to decrease sensitivity. Use of NSAID, PPI, and ASA showed a tendency to affect FC by increasing its sensitivity, PPV, and decreasing the specificity for IBD and OGID.

Organic GI disease (incl. IBD) was found in 39.6% of patients with positive FC, which is in line with previous studies that found an organic diagnosis in 13–37% of primary care patients.^3^, ^4^, ^5^, ^6^, ^25^ In studies by Pavlidis *et al*.^26^ and Turvill,^3^ 3% and 3.6% of patients with negative FC, respectively, were diagnosed with organic GI disease, compared with 9.7% in our study. The results by Pavlidis may be due to a more selected population (primary care patients, aged 18–45 years with suspected IBS) and, by Turvill, the secondary care setting with only referred patients.

Abdominal pain has been shown to be a rather nonspecific predictor of IBD and OGID. Despite the selected primary care population in studies by Pavlidis *et al*.^26^ and Walker *et al*.,^5^ abdominal pain was more common in nonorganic disease. Rectal bleeding and diarrhea were more common in organic conditions,^26^ similar to our study. This is in line with findings by Lasson *et al*.^21^ demonstrating a high diagnostic yield of colonoscopy only for symptoms of bleeding (40%) and diarrhea (31.2%). For onset of IBD, a young age has commonly been described. In the present study, age was not statistically significant and 19% of patients with IBD were >60 years old. An aging population and the increasing incidence of IBD have in recent years been described to contribute to the increased frequency of elderly‐onset IBD.^27^, ^28^

Use of NSAID and PPI was significantly associated with positive FC in our study, and PPI showed the strongest association. This concords with the findings by Lundgren *et al*.^11^ Although minor, accuracy analysis showed a tendency to influence the accuracy measures of FC. As there were few patients on these drugs in the two investigated disease groups, particularly IBD, further studies, taking these variables into account, would therefore be needed to confirm these findings.

In the differential diagnosis of IBD and IBS, according to Otten *et al*., the CalDetect test exhibited a sensitivity of 100%, specificity of 94.5%, PPV of 82.1%, and NPV of 100%, at a cutoff of ≥15 mg/kg.^29^ At a cutoff of 60 mg/kg, the specificity increased to 97.8%, but the sensitivity decreased to 60.6%, which would lead to a substantial loss of IBD diagnoses. In our study, the specificity and PPV at cutoff ≥15 mg/kg were much lower. By increasing cutoff to 60 mg/kg, similarly, the specificity increased and the sensitivity decreased, but to a considerably smaller extent, and a further association of FC with age 35–60 years and male sex was shown. Otherwise, the associations and ORs increased only marginally. These findings illustrate how test accuracy differs depending on how the populations are selected.^3^, ^26^, ^30^, ^31^ In accordance with our results, a recent retrospective study by Freeman *et al*.^18^ found a specificity of 61.5% and PPV of 8.1% at an IBD prevalence of 3.5% in 5970 primary care patients.

The main strengths of the present study are the large, unselected, heterogeneous primary care population and FC testing performed in connection with symptom presentation. FC has been shown to be a useful tool for the assessment and follow‐up of IBD,^9^, ^17^ despite not permitting a positive diagnosis. This study is unique in that it investigates associations between FC and combinations of factors, enabling a positive diagnosis of IBD and OGID, in a large adult primary care population. In addition, it examines the extent to which FC diagnostics may be affected by concomitant treatment with NSAIDs, PPIs, and ASA. Because the GPs did not always proceed with investigation in a case of a negative FC test and therefore could have missed a positive diagnosis, the 5‐year follow‐up is considered to be another strength of this study. Although an even longer follow‐up interval would have been possible, it is less likely that additional diagnoses found would have any association with the FC test outcome.

However, the study also has some limitations. Our data are based on medical records and not patient questionnaires. Hence, there is a risk that GPs did not record the symptoms correctly and some symptoms may not have been included. This, however, seems to reflect daily practice. Hence, the 5‐year follow‐up helped to identify any possible missed cases of IBD or OGID. Data collectors were not blinded for the FC test results, which may have introduced a review bias. The blinding was not possible because the result was presented in the patients' medical records. This, however, should not have altered the collector's interpretation of the index test as the investigations and diagnosis were already recorded by the treating physician. However, a selection bias may have been introduced because only the FC test and no demographic or clinical patients' characteristics constituted the eligibility criteria for this study. Consequently, a rather higher proportion of women in the study cohort, may have potentially resulted in a reduced likelihood in women for IBD and OGID. An additional concern was the lack of reliable information regarding the duration of drug utilization in the medical records.

Furthermore, when planning this study, little evidence of FC testing in general practice was available. The semiquantitative FC analysis, which constituted a base for the present study, has in the meantime been replaced by fully quantitative laboratory‐based tests. Several studies compared various rapid tests with Enzyme‐linked Immunosorbent Assay (ELISA) and found that they compared well across a range of FC levels.^29^, ^32^, ^33^, ^34^ Various FC rapid tests are still in use in other countries, mostly in an outpatient setting.^7^, ^17^, ^35^

## Conclusion

This retrospective study confirms a high NPV of FC in primary care patients ≥18 years with GI symptoms. It reliably rules out IBD and contradicts the presence of other organic GI diseases. A positive FC test together with other predictors, such as diarrhea, rectal bleeding, short duration, or age >35 years should encourage a prioritized investigation. Combining FC with other significant predictors of IBD and OGID increases the specificity and PPV but tends to decrease the sensitivity. Although the likelihood seems to be low, the use of NSAID, PPI, and ASA may affect the diagnostic accuracy of FC for IBD and OGID.

## Supporting information


**Table S1.** Clinical characteristics of participants with negative and positive fecal calprotectin (FC) at cut‐off 60 mg/kg
**Table S2.** Final diagnoses—Other gastrointestinal (GI) inflammatory conditions and Other diagnoses and conditions
**Table S3.** Results of the univariate and binary logistic regression analysis
**Table S4.** Results of the 5‐year follow‐up. The number and valid percentage of patients diagnosed with a new gastrointestinal (GI) disease within 5 years

## Data Availability

The dataset is available from the corresponding author upon reasonable request.
