# Supplementary material for: Diagnostic value of fecal calprotectin in primary care patients with gastrointestinal symptoms: A retrospective Swedish cohort study
Source: JGH Open. 2023 Sep 22;7(10):708–16. doi: 10.1002/jgh3.12972 (PMC10615176; doi:10.1002/jgh3.12972)
Supplement: Supplementary file 1 — Table S1. Clinical characteristics of participants with negative and positive fecal calprotectin (FC) at cut‐off 60 mg/kg Table S2. Final diagnoses—Other gastrointestinal (GI) inflammatory conditions and Other diagnoses and conditions Table S3. Results of the univariate and binary logistic regression analysis Table S4. Results of the 5‐year follow‐up. The number and valid percentage of patients diagnosed with a new gastrointestinal (GI) disease within 5 years [file JGH3-7-708-s001.docx]

**Supplementary Table 1.** Clinical characteristics of participants with negative and positive fecal calprotectin (FC) at cut-off 60 mg/kg.

|  | **FC ≤60 mg/kg, n (%)** | **FC >60 mg/kg, n (%)** | | ***p* values**^†^ | | **OR (95% CI)** ^‡^ | |  |
| --- | --- | --- | --- | --- | --- | --- | --- | --- |
| **Sex** |  |  | | **0.016** | | 0.75 (0.56, 0.99) | |  |
| Male | 273 (32.0) | 171 (38.9) | |  | |  | |  |
| Female (ref.) | 580 (68.0) | 269 (61.1) | |  | |  | |  |
| **Age** |  | |  | | **0.000** | |  | |
| <35 years (ref.) | 362 (42.4) | | 105 (23.9) | |  | |  | |
| 35-60 years | 313 (36.7) | | 151 (34.3) | |  | | 1.44 (1.03, 2.01) | |
| >60 years | 178 (20.9) | | 184 (41.8) | |  | | 3.44 (2.43, 4.88) | |
| **Symptoms** |  | |  | |  | |  | |
| Abdominal pain or discomfort | 559 (65.5) | | 273 (62.0) | | 0.221 | |  | |
| Gases / flatulence / bloating | 252 (29.5) | | 106 (24.1) | | **0.042** | | b | |
| Diarrhea | 314 (36.8) | | 227 (51.6) | | **0.000** | | 2.05 (1.56, 2.70) | |
| Stool consistency fluctuations | 182 (21.3) | | 79 (18.0) | | 0.165 | |  | |
| Constipation | 73 (8.6) | | 28 (6.4) | | 0.189 | |  | |
| Altered stool consistency or form | 84 (9.8) | | 41 (9.3) | | 0.843 | |  | |
| Nausea | 69 (8.1) | | 15 (3.4) | | **0.001** | | 0.37 (0.18, 0.75) | |
| Vomiting | 34 (4.0) | | 17 (3.9) | | 1.000 | |  | |
| Rectal bleeding | 207 (24.3) | | 123 (28.0) | | 0.158 | |  | |
| Weight loss | 119 (14.0) | | 71 (16.1) | | 0.320 | |  | |
| Abnormal clinical findings | 17 (2.0) | | 11 (2.5) | | 0.550 | |  | |
| Family history of GI cancer or IBD | 76 (8.9) | | 40 (9.1) | | 0.918 | |  | |
| **Duration** |  | |  | | **0.000** | | 0.44 (0.33, 0.58) | |
| < 3 months | 194 (22.7) | | 166 (37.7) | |  | |  | |
| > 3 months | 534 (62.6) | | 215 (48.9) | |  | |  | |
| Unclear | 125 (14.7) | | 59 (13.4) | | a | |  | |
| **Concomitant medical therapy** |  | |  | |  | |  | |
| NSAID | 20 (2.3) | | 21 (4.8) | | **0.028** | | b | |
| PPI | 67 (7.9) | | 87 (19.8) | | **0.000** | | 2.49 (1.65, 3.75) | |
| ASA | 35 (4.1) | | 35 (8.0) | | **0.006** | | b | |

Each patient could have reported more than one symptom. OR, odds ratio; CI, confidence interval; GI, gastrointestinal; IBD, inflammatory bowel disease; NSAID, nonsteroidal anti-inflammatory drug; PPI, proton pump inhibitor; ASA, acetyl salicylic acid.

^†^ Univariate analysis performed with Fisher´s exact test, except for Age, which was conducted using Pearson´s chi-squared test.

^‡^ Binary Logistic Regression. The dependent variable is FC.

^a^ Unclear duration was removed from the relevant statistical analysis.

^b^ Statistically non-significant variables removed from the model.

**Supplementary Table 2.** Final diagnoses - Other gastrointestinal (GI) inflammatory conditions and Other diagnoses and conditions.

| **Other GI inflammatory conditions** | | |
| --- | --- | --- |
| Gallstone pancreatitis n=1 | Noninfectious gastroenteritis and colitis n=4 |  |
| Chronic pancreatitis n=2 | Pouchitis n=1 |  |
| Non-specific colitis n=8 |  |  |
| **Other diagnoses and conditions** | | |
| Addison´s disease n=1 | Inguinal hernia n=1 | Secondary amenorrhea n=1 |
| Angioneurotic edema n=1 | Iron deficiency anemia n=4 | Side effects of Enalapril n=1 |
| Anorexia nervosa n=1 | Ischemic enteritis n=1 | Somatoform pain disorder n=1 |
| Cows´ milk allergy n=1 | Kidney stone n=1 | Stress-related illness n=5 |
| Food allergy n=1 | Liver cirrhosis n=1 | Subileus n=1 |
| Hemochromatosis n=1 | Lower urinary tract symptoms n=1 | Systemic lupus erythematosus n=1 |
| Hepatic steatosis n=1 | Monoclonal gammopathy of undetermined significance n=1 | Thalassemia minor n=1 |
| Hepatitis C n=1 | Polymyalgia rheumatica n=2 | Ureteral stone n=1 |
| Herpes zoster n=1 | Primary hyperparathyroidism n=2 |  |

**Supplementary Table 3.** Results of the univariate and binary logistic regression analysis.

|  | **IBD** | **OGID** |
| --- | --- | --- |
| **Variable** | **p value**^†^ | **p value**^†^ |
| Sex (ref. female) | **0.002** | **0.035** |
| Age | 0.688 | **0.000** |
| 35-60 years |  |  |
| >60 years |  |  |
| Symptoms |  |  |
| Abdominal pain or discomfort | **0.000** | 0.503 |
| Gases / flatulence / bloating | 0.352 | **0.005** |
| Diarrhea | **0.000** | **0.025** |
| Stool consistency fluctuations | **0.004** | 0.080 |
| Constipation | x | 0.436 |
| Altered stool consistency or form | 0.813 | 0.186 |
| Nausea | 0.255 | 0.153 |
| Vomiting | x | 0.286 |
| Rectal bleeding | **0.000** | 0.275 |
| Weight loss | 0.418 | 0.107 |
| Abnormal clinical findings | 0.560 | **0.003** |
| Family history of IBD or GI cancer | **0.023** | 0.524 |
| Duration | **0.000** | **0.000** |
| FC | **0.000** | **0.000** |
| NSAID (n=7) | 0.083 | 1.000 |
| PPI (n=43) | 0.816 | **0.004** |
| ASA (n=27) | 1.000 | **0.000** |
|  | **p value**^‡^ | **p value**^‡^ |
| Sex (ref. female) | **0.015** |  |
| Age |  | **0.000** |
| 35-60 years |  | **0.000** |
| >60 years |  | **0.000** |
| Diarrhea | **0.011** |  |
| Stool consistency fluctuations | **0.037** |  |
| Rectal bleeding | **0.000** |  |
| Abnormal clinical findings |  | **0.025** |
| Duration |  | **0.001** |
| FC | **0.000** | **0.000** |

^†^ Univariate analysis performed with Fisher´s exact test, except for Age, which was conducted using Pearson´s chi-square test. x - 0 observations in one group.

^‡^ Binary Logistic Regression. The dependent variables are IBD and OGID.

IBD, inflammatory bowel disease; OGID, organic gastrointestinal disease (excl. IBD); FC, fecal calprotectin. Statistically significant *p* values are in bold.

**Supplementary Table 4.** Results of the 5-year follow-up. The number and valid percentage of patients diagnosed with a new gastrointestinal (GI) disease within 5 years.

|  | **FC <15 mg/kg** | | **FC 15-60 mg/kg** | | **FC >60 mg/kg** | |
| --- | --- | --- | --- | --- | --- | --- |
|  | **n** | **%** | **n** | **%** | **n** | **%** |
| **IBD** |  | 0.0 |  | 0.0 |  | 0.6 |
| Crohn's disease | 0 |  | 0 |  | 1 |  |
| Ulcerative colitis | 0 |  | 0 |  | 2 |  |
| **Other GI inflammation** |  | 1.1 |  | 0.8 |  | 1.2 |
| Gastritis | 3 |  | 0 |  | 1 |  |
| Gastric / duodenal ulcus | 4 |  | 0 |  | 0 |  |
| Gastro-esophageal reflux disease with esophagitis | 0 |  | 0 |  | 2 |  |
| Diverticulitis / diverticulosis | 0 |  | 1 |  | 2 |  |
| **GI tumors** |  | 2.0 |  | 0.8 |  | 2.8 |
| Benign (polyps) | 10 |  | 0 |  | 9 |  |
| Malignant | 3 |  | 1 |  | 3 |  |
| **Functional GI disorders (FGIDs)** |  | 0.2 |  | 0.8 |  | 0.7 |
| Irritable bowel syndrome | 1 |  | 0 |  | 2 |  |
| Dyspepsia | 0 |  | 0 |  | 1 |  |
| Constipation | 0 |  | 1 |  | 0 |  |
| **Other diagnoses in the GI tract** |  | 1.5 |  | 1.6 |  | 3.3 |
| Gallstones | 1 |  | 0 |  | 1 |  |
| Gastro-esophageal reflux disease without esophagitis | 7 |  | 2 |  | 11 |  |
| Miscellaneous macroscopic abnormalities of the gastric mucosa | 1 |  | 0 |  | 1 |  |
| Anorectal disease | 1 |  | 0 |  | 1 |  |
| **Other diagnoses and conditions** | 5 | 0.8 | 0 | 0.0 | 0 | 0.0 |

Malignant GI tumors (FC<15: two small bowel carcinoids, one ureteral cancer metastatic to the liver; FC>15: two pancreatic cancer, one tongue cancer and one small bowel carcinoid). Other diagnoses and conditions (FC<15): Ischemic colitis, fatty liver disease, cirrhosis, allergic and dietary gastroenteritis and colitis, leukoplakia and other disturbances of oral epithelium, including tongue. IBD, inflammatory bowel disease.
